# Design, Implementation, and Validation of a Piezoelectric Device to Study the Effects of Dynamic Mechanical Stimulation on Cell Proliferation, Migration and Morphology

**DOI:** 10.3390/s20072155

**Published:** 2020-04-10

**Authors:** Dahiana Mojena-Medina, Marina Martínez-Hernández, Miguel de la Fuente, Guadalupe García-Isla, Julio Posada, José Luis Jorcano, Pablo Acedo

**Affiliations:** 1 Department of Electronics Technology, Universidad Carlos III de Madrid, 28911 Madrid, Spain; jposada@ing.uc3m.es (J.P.); pag@ing.uc3m.es (P.A.); 2 Department of Bioengineering and Aerospace Engineering, Universidad Carlos III de Madrid, 28911 Madrid, Spain; 100318292@alumnos.uc3m.es (M.M.-H.); 100372912@alumnos.uc3m.es (M.d.l.F.); 100303856@alumnos.uc3m.es (G.G.-I.); jjorcano@ing.uc3m.es (J.L.J.)

**Keywords:** mechanotrasnduction, cell mechanical stimulation, biocompatible piezoelectric stimulator

## Abstract

Cell functions and behavior are regulated not only by soluble (biochemical) signals but also by biophysical and mechanical cues within the cells’ microenvironment. Thanks to the dynamical and complex cell machinery, cells are genuine and effective mechanotransducers translating mechanical stimuli into biochemical signals, which eventually alter multiple aspects of their own homeostasis. Given the dominant and classic biochemical-based views to explain biological processes, it could be challenging to elucidate the key role that mechanical parameters such as vibration, frequency, and force play in biology. Gaining a better understanding of how mechanical stimuli (and their mechanical parameters associated) affect biological outcomes relies partially on the availability of experimental tools that may allow researchers to alter mechanically the cell’s microenvironment and observe cell responses. Here, we introduce a new device to study in vitro responses of cells to dynamic mechanical stimulation using a piezoelectric membrane. Using this device, we can flexibly change the parameters of the dynamic mechanical stimulation (frequency, amplitude, and duration of the stimuli), which increases the possibility to study the cell behavior under different mechanical excitations. We report on the design and implementation of such device and the characterization of its dynamic mechanical properties. By using this device, we have performed a preliminary study on the effect of dynamic mechanical stimulation in a cell monolayer of an epidermal cell line (HaCaT) studying the effects of 1 Hz and 80 Hz excitation frequencies (in the dynamic stimuli) on HaCaT cell migration, proliferation, and morphology. Our preliminary results indicate that the response of HaCaT is dependent on the frequency of stimulation. The device is economic, easily replicated in other laboratories and can support research for a better understanding of mechanisms mediating cellular mechanotransduction.

## 1. Introduction

Cells, as dynamic living beings, have properties that may change according to their own functional states or as responses to external cues. They are subjected to a variety of biochemical and biophysical—in particular mechanical—stimuli. Whilst the role of biochemical signaling pathways have been extensively studied, the relevance of cell mechanics and its alterations in a cell and tissue homeostasis and pathology remain much more unclear.

Thus, over the past few years, attention has been turned towards understanding how mechanical signals can both influence and be influenced by the cell itself and its surrounding environment (during both physiological functions and pathological conditions) [1,2]. Several techniques, devices, and protocols have been ambitiously adapted and developed, not only for measuring cells mechanical properties, but also for mechanically acting on them [3,4,5,6,7,8,9,10,11].

In this sense, many studies have demonstrated that diverse cellular signaling cascades become activated as a consequence of mechanical stimulation, a process known as mechanotransduction (see [12,13] for reviews). Examples of these mechanical stimulations include matrix-elasticity modifications, substrate nanotopography patterns [14,15], local nanoforces (internal or external to the cell) [16,17], and nanovibrations [18,19,20,21,22]. Cells react effectively to such mechanical alterations by inducing cellular responses. These cellular reactions could differ slightly depending on the type of mechanical stimulation [23,24,25]. For example, mechanical stimuli affect cell proliferation and differentiation [26,27,28,29,30]. Other types of responses include variations in global gene expression [31] and upregulation in transcription due to propagation of local stresses (e.g., from the actin cytoskeleton to the nucleus) [32].

Several in vitro studies have relied on mechanical modifications of the substrates on which cells are cultured (e.g., polymer-based hydrogels, two-dimensional pillars, matrix rigidity). Recent research has demonstrated the impact of the mechanical properties of these substrates on cell behavior and reported their use in the measurement of the force that cells exert on them [4,33,34]. However, these methods present inherent shortcomings. Some of the conditions used do not mimic any physiological situation (e.g., nanowire topography patterns) and most of them are not able to actuate dynamically on the cells. Most of these techniques induce only static mechanical changes (e.g., stiffness) and the mechanical conditions the cells face remain constant during the experiments. Furthermore, some studies focus on the mechanical response of the individual cells, which lacks the behavior prevailing in human tissue.

In contrast to the static-based substrate modifications, other studies have evaluated the role of dynamic mechanical stimulations [35], demonstrating that both magnitude and frequency (of the mechanical stimuli) yield changes in the cellular behavior [36,37,38]. Comparing to chemicals, propagation of mechanical signals along the cell hinges on both the physical nature of the stimuli and the physical coupling networks of the cell structure. Under this condition, dynamic mechanical signals can be channeled and propagate through cells much quicker (on the order of microseconds) than release-based chemical signals (in the order of seconds) and thus, not only the cellular responses but timescales of those responses can be influenced [39].

However, the literature on the effects entailing either frequency or amplitude of the mechanical stimulations is limited. Regardless of which are the actual underlying mechanisms, cells sense that sort of stimulation and generate diverse events as a response. In the quest for how cells transduce dynamic mechanical stimuli into chemical and eventually alter their behavior has been stated with two approaches. The first one—purely mechanical—states that dynamic (frequency-dependent) mechanical stimulations can couple to resonant structures of a cell, allowing the stimuli propagates from the extracellular matrix to the nucleus [40]. In the second one (non-exclusive), mechanical stimuli may trigger certain structures/channels that in turn activate in frequency selective ranges, which might raise intriguing possibilities of tuning the frequency of the stimuli to cell response. Overall, a biological cell is an outstanding force sensor and a natural mechanotransducer.

Finally, the possible relevance of the use of mechanotransduction for clinical applications is still to be assessed. Inducing cellular mechanism and/or components as a response to mechanical stimuli to repair damage can gradually emerge as an alternative to classical, medicament-based treatments. For instance, pressure ulcers and chronic wounds lack effective therapies at the present, even though they represent an increasingly prevalent hospital problem [41]. Some studies have applied low-intensity vibration (LIV) and shock wave [42] as a potential therapy for wound healing. LIV in diabetic mice enhanced both granulation tissue and angiogenesis, allowing an accelerating closure of the wounds compared with non-vibrated controls. [43] Local mechanical therapy has been also tested in a rat model of pressure ulcer development [44] and in patients with stage I pressure ulcers [45], sustaining the technique as an emerging alternative for treating wound healing. Although some clinical evidence describing the use of mechanical stimulation is found in literature, the lack of simple platforms that allow in vitro studies for a complete understanding of mechanism mediating cellular mechanotransduction seems to sentence the potential of mechanotherapy.

In this work, we introduce a new device to perform dynamic mechanical stimulation on cell cultures. The device allows a different method to study the response of the cell to mechanical stimulations (i.e., mechanotransduction) by dynamic vibrations. The device is introduced as an instrument to support the study of some complex cellular processes that involve mechanical signals and remain unclear. The device uses a piezoelectric material (whose mechanical characteristics are dynamically modified by means of an electrical voltage) as a cell culture substrate (after electrical isolation and functionalization with collagen), and it is able to induce dynamic mechanical stimuli in HaCaT cell cultures. Preliminary results on the effect of such stimuli in proliferation, migration, and morphology of these cells are also presented.

## 2. Materials and Methods

### 2.1. Description of the Piezoelectric Device System for the Study of the Effects of Dynamical Mechanical Stimuli in Cells

**Figure 1:**
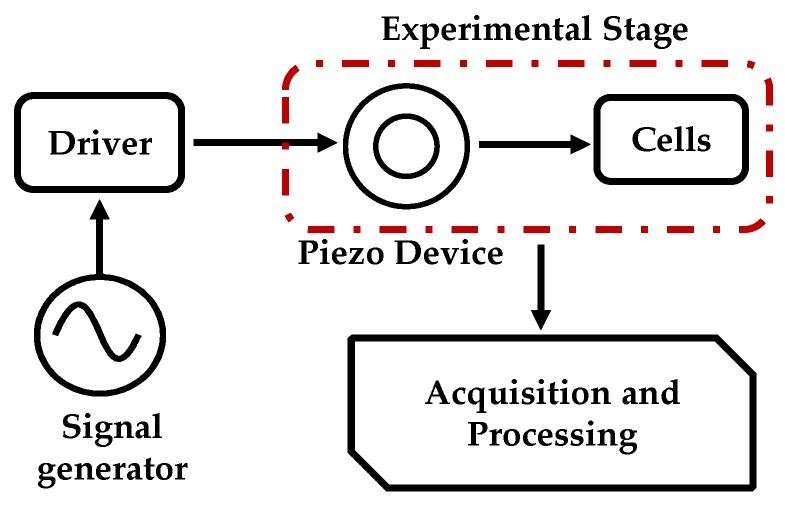
Schematic representation of the completely mechanical stimulator.

Figure 1 schematizes the proposed device to study the effect of mechanical stimuli in cells. The system features an experimental stage (that includes the piezoelectric device and the cell culture), a driver, a signal generator, and acquisition and processing electronics.

The signal generator (Rigol DG4062, Batronix: Preetz, Germany) provides a controlled (both in amplitude and frequency) sinusoidal input to the driver circuit. The driver amplifies the voltage signal that is used to actuate on the piezoelectric device of the experimental stage. The experimental stage is composed of a piezoelectric-based structure interacting with the cells, which were cultured within the confinements of the piezoelectric actuator, as is described in the following section.

#### 2.1.1. Experimental Stage

**Figure 2:**
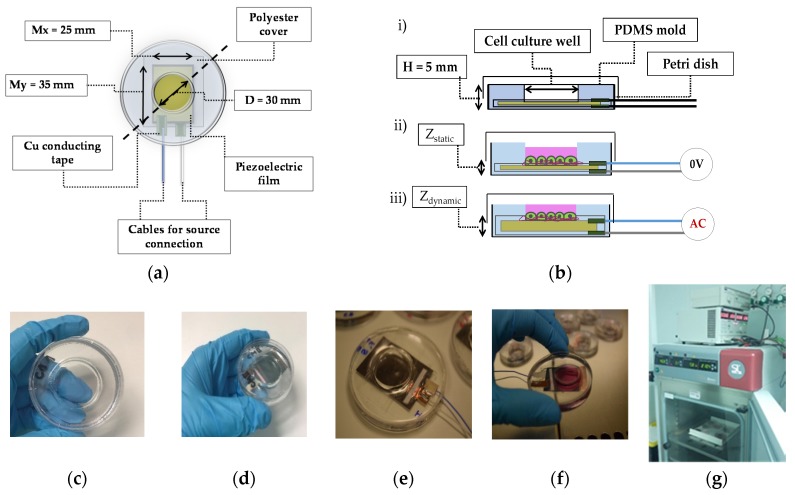
(**a**) description of the conformational domains in the experimental stage based on a PVDF actuator; (**b**) lateral view of the schematic representation shown in (**a**). (i) the PVDF film is in the bottom of the Petri dish and a PDMS mold achieves the cell culture well. (ii) and (iii) show a cell culture with basal medium and cell cultures confined within the PDMS mold and with the PVDF film as a substrate (under static condition (ii) (0 voltage in the electronics stage) and dynamic condition (iii) (AC voltage in the electronics stage); (**c**) control set for all the experiments; (**d**) control set of the piezo device for static conditions; (**e**) dynamic set for cell culture stimulation at a determined frequency and amplitude (it is noted the external cable connections); (**f**) handling cell cultures on the piezoelectric devices inside a biosecurity cabin; (**g**) the piezoelectric system containing the HaCaT cells is placed inside the cell culture incubator with a lateral hole to allow electrical access (a signal generator, power supply, and the driver is observed outside the incubator).

The experimental stage is composed of a piezo-actuator, a PDMS (polydimethylsiloxane) mold, and a Petri dish. The piezo actuator is a PVDF (polyvinylidene fluoride) [46] film of 28 μm in thickness polarized in its transverse axis (CAT-PFS0003, TE Connectivity, Schaffhausen, Switzerland, 80 mm × 110 mm). The PVDF film is coated with Ni-Cu metallized layers; however, no external electrodes were presented for connection or any insulation layers. A copper adhesive tape (3M™; 1181-12, 3M: St. Paul, MI, USA) is used to connect the cables from the driver to the metallized layer. The adhesive is conductive, connecting both elements: the metallic faces of the PVDF and the tape with the soldered cables. The original PVDF film was split in 25 mm × 35 mm films fitting within the Petri dishes specifications (see Figure 2a,b).

The piezoelectric films with the attached wires were covered with a single-sided adhesive polyester film (3M™, 58 μm in thickness). This non-conductive polymer succeeded in preventing electrical stimulations of the cell culture while transmitting the reverse piezoelectric effect.

The piezoelectric together with the electrodes were placed within a modified P60 polystyrene Petri dish (Ø 60 mm., PDIP-06N-500, LabBox: Barcelona, Spain). The opacity of the piezoelectric material (Figure 2f) makes it impossible to look at the specimens through a transmission light microscope, requiring the use of an inverted fluorescence microscope (Olympus BX53, Olympus Inc., Tokyo, Japan) with limited vertical space to place the experimental stage. For this reason, the height of the dishes was reduced to 15 mm by using mechanical tools (Dremel^®^ 8100 Model, Dremel^®^, Racine, WI, USA). The cables exit through holes (2 mm in diameter) on the *z*-axis of the petri dish.

The isolated piezo-actuators and cables (inside modified dishes) were embedded into molds of a biocompatible elastomer, poly-dimethylsiloxane (PDMS) (Sylgard^®^ 184, Dow Corning, Midland, MI, USA). This elastomer is widely used in the microfluidic field [47,48]. It was molded leaving a circular exposed PVDF space of 30 mm diameter in which the cell cultures were performed (Figure 2a,b). The already prepared PDMS (see PDMS section for the preparation process) was poured and it was cured at 70 °C during one hour in the oven (Model 100-800, Memmert GmbH, Scwabach, Germany).

Several devices were fabricated, and some resulting experimental stages are depicted in Figure 2c–f. Devices used as controls were produced only with the PDMS pouring stage (Figure 2c), lacking the piezoelectric membrane. In those devices, the cells were seeded directly on the polystyrene Petri plate surface within the confinement of the PDMS ring. The static (Figure 2d) and dynamic (Figure 2e) setups were similar except that the static controls (named CM for control material) were not electrically stimulated; hence, no holes were performed in the plates to take out the cables. This reduced possible contaminations and culture medium leakage. The dynamic setups (named F-1 Hz and F-80 Hz for the frequency at 1 Hz and 80 Hz) were electrically stimulated by means of an electronics stage (i.e., driver and a signal generator). The electronic stage is properly configurated for each experiment by setting the frequency and voltage amplitude to the driver from the signal generator, and the cables from the driver stage are introduced into the incubator through a lateral hole (Figure 2g).

Cells in culture medium were plated within the PDMS ring inside a cell culture laminar flow cabin and cultured in a cell incubator as described in Methods.

#### 2.1.2. Collagen Functionalization

For functionalization of the mechanical actuator surface, 2.5 mL of collagen solution (Sigma Aldrich, 0.20%, Type II from Calfskin, Sigma Aldrich, St. Louis, MO, USA) (Solution at 1:20 *v/v* in PBS) was added to each well. They were left exposed to UV radiation for 2 h inside a biosecurity cabin. The remaining solution was removed, and samples were washed with PBS twice.

#### 2.1.3. PDMS Curing Process

The PDMS fabrication kit (Sylgard^®^ 184, Dow Corning, Midland, MI, USA) contains a base and a curing agent. The relation between base and curing agent generates difference in the rigidity of the final elastomer. The proportion used was a ratio of 10:1 base-curing agent.

Both components were weighted in an analytic balance (Ohaus Voyager, Ohaus, Nänikon, Switzerland). Manual stirring continued until a homogeneous mixture was obtained after adding the two components. Air bubbles at the mixture obtained were eliminated using a vacuum chamber. The mixture liquid is next poured onto the master mold. Finally, for curing, they were introduced in a preheated oven (Model 100-800, Memmert GmbH, Schwabach, Germany) at 70 °C for one hour.

#### 2.1.4. Driver

A driver circuit was designed to amplify and condition the signal required at the piezo-actuator input. The driver stage was based on power operational amplifiers configured in a bridge configuration. The pair of amplifiers used (PA79, Apex Microtechnology, Tucson, AZ, USA) provided the output voltage, in which one acted as a master and the other one as a slave.

The overall gain of the amplification stage is +20. The gain is fixed by the master, while the slave amplifier is set at unit gain. The power supply (IHB200-0.12, International Power, London, UK) bias the two amplifier modules. The schematic circuits and the printed circuit board (PCB) were implemented in Altium Designer software (Altium Limited, Chatswood, NSW, Australia).

### 2.2. Cell Culture

Fluorescent HaCaT cells (see [49] for details of the cell line), a line of immortalized human keratinocytes, were provided from CIEMAT (Centro de Investigaciones Energéticas Medioambientales y Tecnólogias, Madrid, Spain). They were transformed by a GFP (Green Fluorescent Protein) expressing a retroviral vector to enable cell culture observation by fluorescence microscopy. Cells were kept cryopreserved at −83 °C in culture medium containing 10% (*v/v*) dimethyl sulfoxide (DMSO, Sigma Aldrich).

For passaging, HaCaT cells were defrozen and cultured in polystyrene dishes P100 (100 mm diameter, 78 cm^2^, ThermoFisher Scientific) in basal medium (Dulbecco’s Modified Eagle Medium 1.8 Mm Ca^2+^, DMEM, ThermoFisher Scientific) supplemented with 10% fetal bovine serum (FBS) and 2% (*v/v*) penicillin-streptomycin (Ab, 100,000 U/mL–10,000 μg/mL, respectively). They were cultured at 37 °C, 38% humidity, 5% CO_2_ in a cell incubator (Shel Lab CO_2_ Serie; Sheldon Mfg. Inc., Cornelius, OR, USA). The culture medium was changed every 2–3 days. At 80% confluence (condition assessed by microscopy and experimentally), cells were trypsinized (0.25%-EDTA) and plated at a 1:4 dilution.

For cytotoxicity and proliferation assays (see below), 2 × 10^5^ cells (2.8 × 10^5^ cell/cm^2^) in 2.5 mL of the supplemented DMEM medium above described were seeded in the PDMS rings of the piezoelectric devices and cultured for 0, 1, 3, and 6 days.

### 2.3. Cytotocicity and Proliferation Assays

The AlamarBlue^®^ (AB) assay was used for both testing any cytotoxic effect of designed device on the cells and assessing cell proliferation. This assay is an indicator of cell metabolic activity and viability based on the dye indicator resazurin. Viable cells reduce resazurin to resorufin generating a fluorescent signal. An increase of AB over time is often used as an indicator of proliferation [50].

The AB assay is explained in detail in [51]. In summary, the culture medium was substituted for Alamar Blue rezasurin (BUF012A, Bio-Rad AbD Serotec Ltd., Hercules, CA, USA) diluted in 1:10 phenol-red-free medium (D5030, Sigma). 2.5 mL of this AB reactive medium was added to each sample. After 4 h, the AB medium was stored and labelled into 96-well plates for absorbance analysis (a microplate reader ELISA, Clariostar, BMG Labtech, Ortenberg, Germany).

The absorbance was measured at 570 nm and 600 nm. The absorbance reduction percentage (PR%) indicative of metabolic activity, is calculated by applying the formula (1):

(1) $$\mathrm{PR}\left(\%\right)=\frac{\left(\varepsilon_{\mathrm{ox}}\right)\lambda_{2}{\cdot A\lambda }_{1}-\left(\varepsilon_{\mathrm{ox}}\right)\lambda_{1}{\cdot A\lambda }_{2}}{\left(\varepsilon_{\mathrm{red}}\right)\lambda_{1}{\cdot A\prime \lambda }_{2}-\left(\varepsilon_{\mathrm{red}}\right)\lambda_{2}{\cdot A\prime \lambda }_{1}}$$

where $\varepsilon_{\mathrm{ox}}\;$ and $\varepsilon_{\mathrm{red}\;}$ are the molar extinction coefficients of oxidation and reduction forms of AB, respectively, A is the measured absorbance of the samples, A’ is the absorbance of the negative control (for the negative control, 2.5 mL of AB reactive medium was added to sterile flask without cells), λ_1_ is the wavelength of 570 nm, and λ_2_ the wavelength of 600 nm.

The percentage reduction (PR%) were normalized using the average value obtained for the control devices at the last day of the experiment prior statistical analysis.

### 2.4. Migration Assay

#### 2.4.1. Wound Generation

For assessing cell motility, we used two methods to generate an artificial wound in the cell monolayer. The first one was a scratch assay using a micropipette tip [52]. In the second method, the size and position of the wound were fixed placing on the culture plate a handmade PDMS stencil. This last method prevents potential cell damage at the wound edges produced by scratching.

Scratch wounds were performed by seeding in the culture well of the device and controls (see Figure 2c–e) 1.44 × 10^6^ cells (2.03 × 10^7^ cell/cm^2^)—a number experimentally determined to generate a confluent monolayer—in 2.5 mL of culture medium overnight. The next day, the cell attachment to the substrate and monolayer formation were assessed by microscopy. Afterwards, a p100 micropipette tip (LabBox, Ø 1.2 mm) was used to generate an approximately a 500 μm wide artificial wound in the monolayer. After scratching, the cells were washed twice with PBS at 37 °C to remove cellular debris. They were then fed with 2.5 mL fresh media. Time 0 of the experiment was taken at this moment.

For the second method, a stencil (500 μm wide) was inserted in the middle of device culture well. Equal numbers of cells were seeded at both sides of the stencil. After 24 h, the stencil was carefully removed and monolayer formation were checked by microscopy. This moment represents time 0 in the experiment. Immediately after wound generation using any of the two methods, the piezoelectric devices were activated (see the Results section).

Images for monitoring the width of the bound were taken at 0, 12, 24, and 28 h. Enough sample fields were acquired to cover the entire wound using a BX53 Digital Fluorescence Microscopy, a GFP filter (excitation/emission 395/503 nm) and a magnification of 20×.

#### 2.4.2. Image Processing

Wound healing was analyzed by measuring the width of the scratch over time at several places. Widths were normalized to 0 time for each wound.

Triplicate samples (control devices (C), devices without stimulation (CM) and devices with stimulation (3 for frequency of 1 Hz and 3 for 80 Hz)) were used in each experiment.

The area reduction due to migration was determined according to Formula (2):

(2) $$\mathrm{Migration}\;\mathrm{area}\;\mathrm{reduction}=\frac{A_{t}}{A_{0}}$$

where $A_{0}$ is the mean initial area and $A_{t}$ is the mean area within the wounds’ boundaries at time t.

### 2.5. Scanning Electron Microscopy

Scanning Electron Microscopy (SEM) was used to study cellular topography. To this, samples were fixed with 2.5% (*v/v*) glutaraldehyde in distilled water (Sigma Aldrich) at room temperature for 1 h and later washed three times with PBS. Thereafter, samples were dehydrated by passing them through 30% ethanol (5 min), 50% ethanol (5 min), 70% ethanol (5 min) and 100% ethanol (5 min). Samples were air dried in the biosecurity cabins and gold was sputtered on the material to form a 20 nm thick layer on the surface (high vacuum sputter coater Leica EM ACE600, Leica Microsystems, Weitzlar, Germany).

In addition, 20 × 10^4^ cells in 2.5 mL of culture medium were seeded in culture media in two independent experiments. Each repetition consisted in control devices (C), static devices (CM), stimulated devices with 1 Hz (FR1Hz) and 80 Hz (FR80Hz). Twenty-four hours after seeding, the dynamic devices were firstly stimulated with frequencies of 1 Hz and 80 Hz, respectively, and samples were fixed after 48 h of continuous stimulation.

### 2.6. Actin Fluorescence and DAPI

After 24 h and 48 h of stimulation, cells were rinsed with PBS twice and fixed in 4% (*v/v*) paraformaldehyde in PBS for 20 min at room temperature. After permeabilization with a 0.1% (*v/v*) Triton X-100 (Sigma Alderich) in PBS for 15 min and blocking in 1% (*v/v*) BSA in PBS for 30 min, 1 mL of fluorescent phalloidin was added in the samples (1:1000 *v/v* in blocking solution, Toxin Phalloidin iFluor 555, Abcam) for 90 min. Finally, to visualize cell nuclei, samples were washed sequentially in PBS and 2 mL of DAPI (1:1000 *v/v* in PBS, DAPI 1mg, Sigma Aldrich) was added for 10 min. After the last wash in PBS, the samples were carefully mounted using mounting medium (M1289, Sigma Aldrich) for microscopy visualization.

#### Size of the Nucleus

To analyze quantitively the size of the nucleus, DAPI images were processed using ImageJ software. The nucleus was segmented and fitted to an ellipse, in which the length and width of the nucleus correspond to the long and short axes of the ellipse, respectively. Histogram of the values of long axes was further fitted to a log-normal distribution using Origin software. The average size of the nucleus was determined according to Formula (3):

(3) $$\langle {\;D}_{\mathrm{mean}}\rangle ={\;D}_{0}\cdot e^{-(\frac{\sigma^{2}}{2})}$$

where $D_{0}$ is the center of the distribution of the fitting, and σ is the standard deviation in the fitting.

The average sizes in the experimental groups were normalized using the average value obtained for the negative controls at that specific time (i.e., at 48 h). The normalized ratios were compared by a grouped analysis two-way ANOVA followed by a Bonferroni post-test. A confidence interval of 95% was applied and the collected *p*-values were examined. Graph represents the normalized average size of the nucleus and the standard deviation of the size calculated by the image processing algorithm.

### 2.7. Microscopy

For microscopy observation, a BX53 Digital Fluorescence Microscopy (Olympus Inc., Tokyo, Japan) equipped with a CCD camera (Olympus DP26) and analySIS-getIT software (Olympus^®^) was used. Appropriate filters were used for each fluorophore in fluorescence experiments (DAPI excitation/emission 390/450 nm and TRITC excitation/emission 543/569 nm). For image analysis and processing, both ImageJ (free download from the NIH using a macro for wound healing) and Matlab with an Imaging Processing Toolbox were used.

### 2.8. Statistical Methods

For statistical analysis, a grouped analysis two-way ANOVA was implemented by GraphPad Prism. As each assay and controls were sampled five times of separated one, five estimates of standard errors of the repetitions were obtained for each treatment through post Bonferroni corrections. By this, each group of samples was compared to all the other samples and *p*-values were examined. *p*-values higher than 0.05 were not considered as significant differences between samples and confidence interval of 95% were used.

Results from the statistical analysis were depicted in the figures in the form of asterisks, the higher number of symbols being the higher significance between the samples and the control values.

In the cytocompatibility and proliferation assays, the mean and the standard deviations of the samples are represented. The reduction percentages (*y*-axis) are normalized to the maximum value reached by the negative control at end days. Initial zero values do not correspond with a 0% AB reduction at the start in all samples, but every culture was subtracted its initial reduction percentage for setting a common reference point in the origin.

Regarding migration, the wounds’ area values were normalized to the area at time 0—the initial area of the wound—so that the area reduction at different times is expressed as a percentage of the initial area. The normalized values were analyzed with a linear regression fit with a confidence interval of 95%.

## 3. Results

### 3.1. Characterization and Validation of the Mechanical Properties of the Device for Dynamic Mechanical Studies

#### 3.1.1. Laser Interferometry

**Figure 3:**
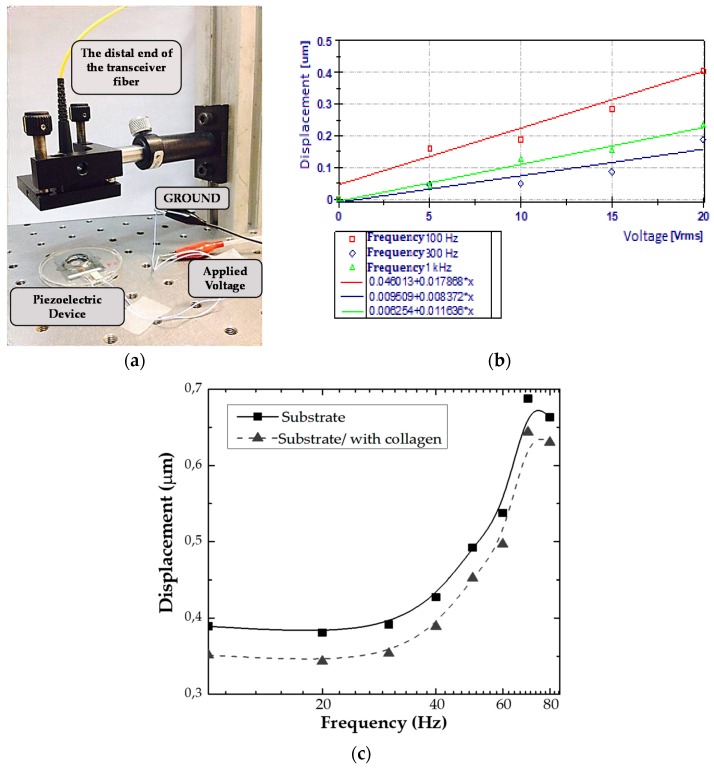
(**a**) interferometric layout for analysis of the piezoelectric device; (**b**) plot showing a linear relation between piezo actuator input voltage and output amplitude for several frequencies; (**c**) response curve of the PVDF (mechanical deformation) vs. the applied frequency in the device. The curve shows negligible differences between the response of the PVDF functionalized with collagen and without functionalization.

The dynamic mechanical properties of the devices were characterized by an interferometric system. The interferometric system measured the vibration amplitude response versus the stimulation frequency of the mechanically active PVDF substrate on which cells were cultured. The laser interferometer employed was a common path fiber optical topology (CPT), previously reported in [53]. The CPT is created by an optical cavity between the surface of the device and the distal end of the transceiver fiber (Figure 3a). The cavity (distance between the fiber and the target) was set at 4 cm. The system was illuminated with a laser diode (990 nm, Throlabs, Newton, NJ, USA), which was chirped with a sinusoid of 500 kHz frequency. The quadrature components in the phase measured are recovered applying a phase generated carrier. To perform the experiments, a LabVIEW (National Instruments, Austin, TX, USA) controlled PXI DAQ was used.

The setups were tested in two conditions: right after fabrication, and once functionalized with collagen (last reconditioning before cell seeding). A piece of reflecting paper was attached at each measurement point to improve the reflectivity. The acquired signals were filtered and post-processed (DIAdem, LabVIEW). This way, the frequency and magnitude responses of the actual mechanical displacement of the PVDF substrate were obtained.

The devices were characterized at voltages from 2 to 20 V_RMS_ and frequencies from 10 Hz to 90 Hz, which are below the first natural frequency shown in Appendix A. A linear and consistent tendency was observed by increasing the amplitude of voltage applied (Figure 3b). The dynamic response (Figure 3c) showed very small changes in the mechanical behavior of the devices functionalized with collagen compared with those without functionalization.

#### 3.1.2. Physical Analysis and Estimation of the Applied Forces

Understanding the effects of the mechanical stimulations on cells presents a challenge in discerning the key parameters conducing to the cellular responses. Some specific variables of the mechanical systems which can target the cellular processes include amplitude, frequency, and the resultant acceleration. However, the force may be a critical factor; even in the case of two similar accelerations, the system could exert different forces (the accelerative force depends on Newton’s second law). For this reason, an analysis to assess the actual force acting on the cells associated with the dynamical (sinusoidal) stimulus follows.

Since in our experiments we used the same amount of medium and the same cell line (same surface area), and cells are adhered to the oscillating substrate surface (active piezoelectric substrate) prior to stimulation (cells are self-sustaining under gravitational force (g)), we only consider the forces exerted due to the peak acceleration of the piezoelectric device, that is, the apparent accelerative forces.

The mechanical displacements of the system follow a sinusoidal motion. In this case, it is possible to estimate the accelerative force, knowing the amplitude and the angular frequency exerted (the product of the amplitude A and the square of the angular frequency). The peak force perceived by each cell attached to the substrate can be estimated by considering the second Newton’s law, expressed in (4), where F is the vector sum of the force, m is the mass and a the acceleration:

(4) $$F_{\mathrm{Newton}}{=\mathrm{ma}=m}_{\mathrm{cell}}\ddot{x}$$

Knowing that the acceleration in (4) is produced by the sinusoidal output in the given actuation system, the expression for $\ddot{x}$ is obtained based on the displacement (5) done by the piezoelectric film, where A corresponds to the amplitude of the waveform (measured in meters) and ω refers frequency of the sinusoidal signal:

(5) $$a=\ddot{\;x\;}=\frac{d^{2}x}{{\;\mathrm{dt}}^{2}}{=-A\cdot \omega }^{2}\cdot \sin(\omega t)$$

Returning to the calibration of the device shown in Figure 3c, 1 Hz frequency corresponded to a 0.4 µm displacement peak-to-peak; meanwhile, the 80 Hz frequency gave around a 0.7 µm peak-to-peak displacement. This gives an acceleration of approximately 1.2 × 10^−5^ m/s^2^ and 0.18 m/s^2^, respectively. The estimation of the apparent acceleration is then 1.22 × 10^−6^ g and 0.02 g, respectively.

In this case, the apparent forces being transmitted through the attached cell can be obtained by considering the apparent acceleration generated by the substrate and the cellular mass (the density of endothelial cells is considered to be 1.03–1.05 g∙cm^−3^ and the mean cross sectional area to be 30 µm × 30 µm). For stimulation at 1 Hz and 80 Hz, this results in forces of approximately 0.04 pN and 0.8 pN, respectively. The reported forces associated with cellular processes have a magnitude in the order of nano to piconewtons [54], whereby the resultant accelerative forces induced in the cells by the device are on the same order of magnitude. Under our consideration, with the use of the device, the cells will not cease cellular activity. If higher forces had been applied by the system, apoptosis and/or cell detachment from the substrate could have been induced.

Finally, the absence of fluid streams induced by the vibrating substrate was confirmed to properly attribute the observed results to mechanical stimuli and not because of flows within the structure. This is important, as steady streaming may result within the confinements of the device from the action of an oscillatory non-conservative body force (the sinusoidal wave that it is being applied) [55].

To that extent, substrates were filled with water, and Fluorescein Sodium (C.I. 45350, Panreac, Darmstadt, Germany) was added dropwise under blue light to enhance visualization. The non-existence of fluxes was monitored visually by the absence of tracks once the yellow fluorescein drops contacted the aqueous solution. The yellow characteristic fluorescein sodium added to the water solution was rapidly homogenized within the confinements of the device. No fluxes were checked in the dissipation process, but a gradual diffusion of the dye. The diffusion proceeded in the same fashion in both the static and dynamic devices.

### 3.2. Preliminary Studies of the Effects of Dynamic Mechanical Stimulation Using the Proposed Device on Cell Proliferation, Migration, and Morphology

After the description and characterization of the instrument in previous paragraphs, and in order to demonstrate the utility of such instrument to study mechanical stimulation in cell culture studies, we performed a set of experiments using a cell monolayer of an epidermal cell line. These preliminary results follow.

#### 3.2.1. Selection of Dynamics Conditions for Cellular Experiments

All cellular experiments were assessed under same dynamics conditions. As per vibration curve (Figure 3c), the displacement of the active substrate peaks at frequencies of 80 Hz. The value is 0.7 μm compared to 0.4 μm at low frequency. Therefore, conditions selected were 1 Hz frequency due to similarity to biological frequencies in the human body [18,56,57] and 80 Hz frequency because of this maximum displacement of the substrate. The vibration duration was applied continuously during the experiment (i.e., six days for proliferation assay; 30 h for migration; 72 h for SEM micrographics, and 48 h for immunostaining).

#### 3.2.2. Cytocompatiblity and Proliferation Assays

The viability of the devices was assayed in a total of *n* = 28 control samples (C) and *n* = 25 control material samples (CM) for a total of eight different experimental repetitions.

**Figure 4:**
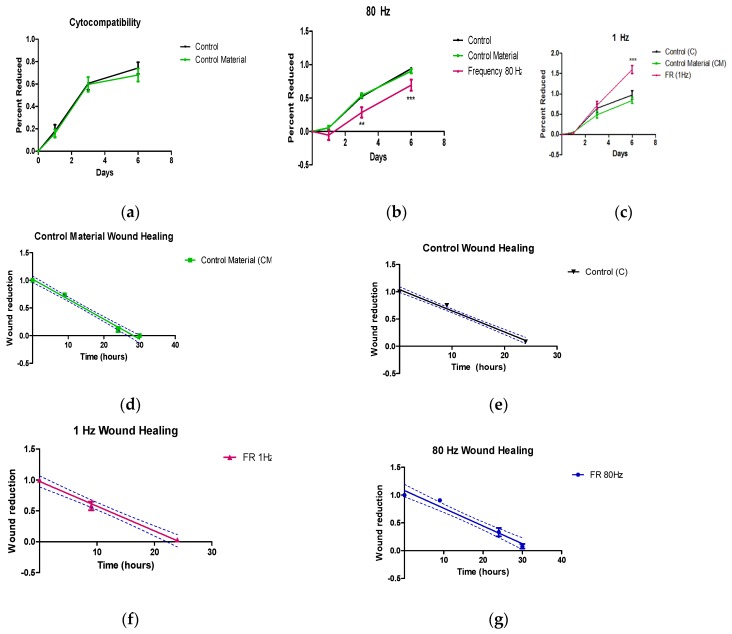

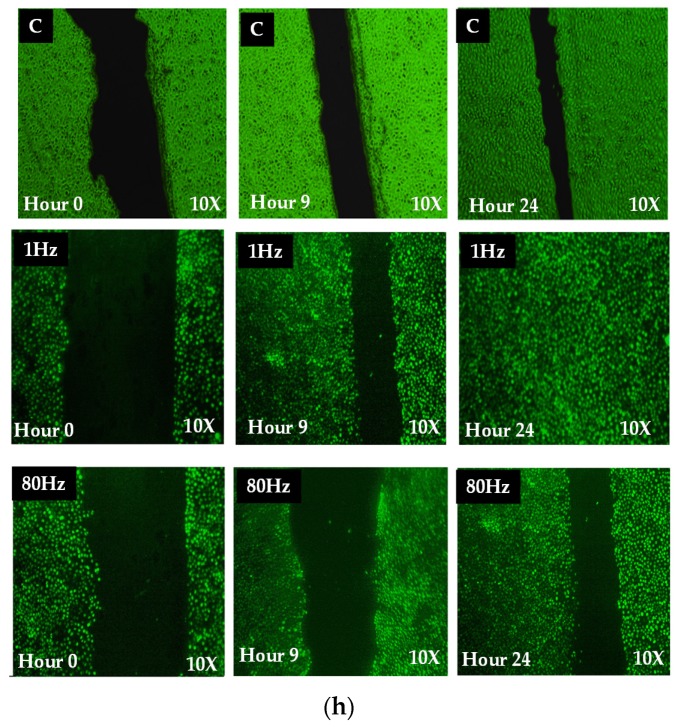
(**a**) curve of the cytocompatibility assay for eight experiments repetition (*n* = 28 control samples (C) and *n* = 25 control material samples (CM)). No significance (*p* < 0.05) was found. (**b**) curves for experiment 80 Hz applied. (*n* = 9 control samples (C), *n* = 9 control static samples (CM) and *n* = 8 dynamic samples at 80 Hz frequency) ** Significance (*p* < 0.05) in days 3 and 6. (**c**) curves for two repetitions of experiment 1 Hz applied (*n* = 12 control samples (C), *n* = 12 control static samples (CM) and *n* = 12 dynamic samples at 1 Hz frequency) *** Significance (*p* < 0.05) in days 6 and 8; (**d**) area decrease in scratch assay due to migration process in control devices (C). Value in the curve at 30 h represents that the healing closed completely at the observation, but not the closing time.; (**e**) area decrease in scratch assay due to migration process in control. devices with piezoelectric material without stimulation (CM); (**f**) area decrease in scratch assay due to migration process in piezoelectric device with 1 Hz frequency stimulation (FR 1 Hz); (**g**) area decrease in scratch assay due to migration process in piezoelectric devices with 80 Hz frequency stimulation (FR 80 Hz); (**h**) wound progression in a control (C) device, and experimental groups with 1 Hz and 80 Hz stimulation frequency at different times.

Cytocompatiblity results are depicted in Figure 4a. Curves represent AlamarBlue^®^ reduction percentages and correspond to the mean and standard deviations of the samples of each study group. The proliferation rate is related to the slope of each curve, respectively.

In this study, no significance between static piezoelectric device and negative control was detected (*p* < 0.005). The proliferation rate in both attains up reaching almost the same growth-related metabolic activity. Given that the cellular culture of the piezoelectric device follows a constant proliferation and no statistical significance attaining in reduction values the negative control, it can be stated that the designed devices are biocompatible.

Figure 4b shows the proliferation and growth in 80 Hz group. A total of *n* = 9 control samples (C), *n* = 9 control static samples (CM) and *n* = 8 dynamic samples at 80 Hz frequency (FR (80 Hz)) were tested in a total of three experimental repetitions. The FR (80 Hz) samples were metabolically less active—i.e., less proliferative—being a difference of 15% at day 1, 17% at day 3, and 8% at day 6.

The results of 1 Hz experiments are detailed in Figure 4c which correspond with the results pooled from three identical experiments. A total of *n* = 12 control samples (C), *n* = 12 control static samples (CM) and *n* = 12 dynamic samples at 1 Hz frequency (F (1 Hz)) were tested. The proliferation rate of the negative controls performed in the static piezo devices and Petri dish is significantly lower than any culture taking place in the dynamic piezoelectric actuating device. Up to the third day, all samples showed similar reduction percentage, but 1 Hz samples still tilted to the upside and reached the highest level of metabolic activity on the sixth day.

#### 3.2.3. Migration Assay

The migration experiments involved a total of *n* = 9 control samples C, *n* = 9 control material samples (CM), *n* = 9 FR (80 Hz) and F1 (1 Hz) in a total of three different experimental repetitions.

Figure 4d–g shows the normalized scratch’s area closing in the samples. Confidence bounds are shown in discontinuous lines. Figure 4d depicts the characteristic plot in control material samples. Value in the curve at 30 h represents that the healing closed completely, but not the closing time. Thus, predicting the healing time used the linear regression fit of GraphPad Prism.

A summary of all the obtained parameters can be found in Table 1. Linear regression of wound healing profiles altogether showed that the differences between the elevations or intercepts (i.e., *X*-intercept when *Y* = 0.0 and thus closure time) were extremely significant between the stimulated samples at 1 Hz and 80 Hz and the control specimens (*p* < 0.0001).

Samples stimulated at 1 Hz (Figure 4f) significantly migrated at a faster rate to close the scratch in comparison with the control sample. In this group (1 Hz), the healing process is completed at 24.41 h. The ones stimulated at 80 Hz significantly decreased this rate compared with controls, as can be observed in the normalized migration’s area closing (Figure 4g). The wound advancement closed around 34 h. The cells grown onto the piezoelectric device without stimulation did not show any significant deviation compared with control samples (see Table 1). An example of the wound progression in a control (C) sample and experimental groups with 1 Hz and 80 Hz stimulation frequency is shown in Figure 4h, where the wound progression at hour 0, hour 9 and hour 24 are shown using the microscope.

#### 3.2.4. SEM Visualization

**Figure 5:**
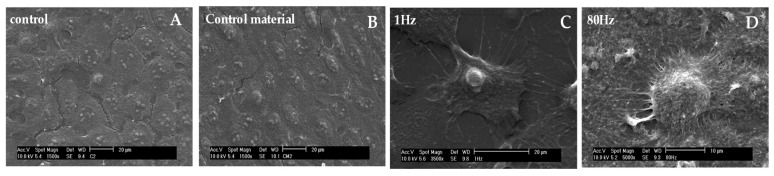
Electron microscopy images of samples under different conditions. The micrographics show difference in surface area and projections after stimulation. Non-significance is observed between controls (**A**) and static controls (**B**) at 48 h of observation. The morphology is clearly different between control groups (control (**A**) and control material (**B**)) with 1 Hz samples (**C**) and 80 Hz samples (**D**). The 1 Hz stimulated group cells show cytoplasmic projections and condensed nucleus at 48 h. The 80 Hz stimulated group shows cells with rounded shape at 48 h. Length scale: 20 µm.

The cell topography for different cases was observed using a scanning electron microscope (SEM) and there are depicted in Figure 5.

The SEM picture of a HaCaT cells grown in a control device, Figure 5A, shows cells with a polygonal shape and collective grown in colonies. The cell’s diameter is about 35 µm. The control samples seeded on static device (Figure 5B) exhibit similar topography to the cells grown in the control. These epithelial cells end to form colonies as they did in the control devices (Figure 5C), well attached to the surface. The similarities in morphology between control (cells seeded on Petri dishes) and control devices (static conditions) persist along the experiments (i.e., during 48 h). (Figure 5A,B). The morphology is clearly different between control groups (control and control material (Figure 5A,B) with 1 Hz samples (Figure 5C) and 80 Hz samples (Figure 5D). The 1 Hz stimulated group cells show cytoplasmic projections and condensed nucleus at 48 h, while the 80 Hz stimulated group show cells with rounded shape.

#### 3.2.5. Immunostaining

**Figure 6:**
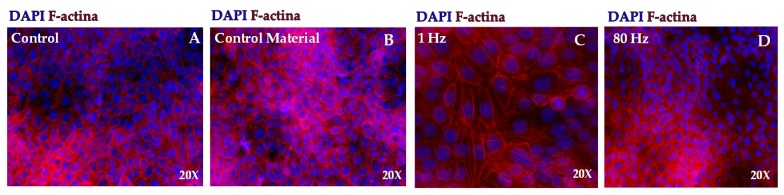
Immunostaining images of f-actin and DAPI at 48 h after continuous stimulation. Changes in actin distribution is observed between control samples (control (**A**) and control material (**B**)) with 1 Hz stimulated samples (**C**). In the 80 Hz group (**D**), significant changes compared with control are not observed. Nucleus staining does not show significant difference in size in control samples compared with 80 Hz stimulated samples. In the 1 Hz group (**C**), in some fields, the nucleus sizes were oval and elongated consistent with directions of the stress fiber.

To study further the morphological changes shown in the SEM pictographs, an immunofluorescence study was also performed. Figure 6 depicted the actin cytoskeleton staining for 1 Hz and 80 Hz excitation frequency group and a control groups (cells seeded on glass coverslips and static control).

Furthermore, 1 Hz stimulated samples showed the f-actin stress fibers thicker and more elongated compared with control samples after 48 h of stimulation (Figure 6C). These cells maintained apparent cytoplasmic projections (filopodia), a more prominent spatial spreading, and a clearly dispersed stress fiber. Besides this, f-actin filaments appear distributed in the pericellular spaces.

In control samples, the f-actin presented less tension and projection compared with stimulated samples. Rather, f-actin appeared distributed at the borders of the cells and showed neither elongation nor orientation. In the 80 Hz samples, the actin protein was observed without significance changes compared with controls.

**Figure 7:**
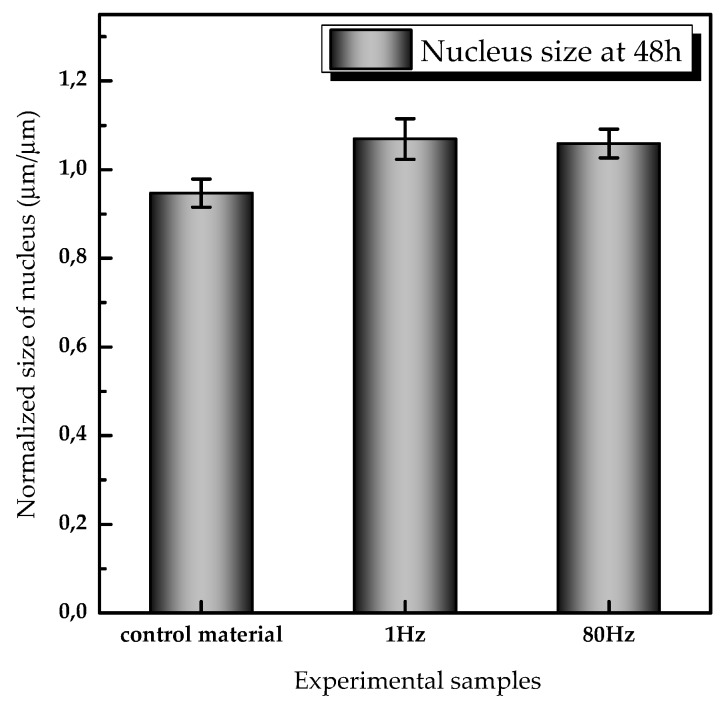
Average size of the diameter of nucleus normalized to control samples at 48 h after continuous mechanical stimulation (number of nuclei analyzed > 300 for both control and experimental groups).

Nucleus staining illustrates a slightly increasing in size under 1 Hz dynamic conditions (Figure 6C and Figure 7). After 48 h of continuous stimulations, the nucleus sizes in the 1 Hz group was oval and elongated consistent with directions of the stress fiber and varying the size by 0.069 units compared with controls; in 80 Hz, the average size of nucleus was 0.059 units greater than controls. In control static samples, the nucleus remains similar in size compared with controls. The nucleus did not show a significant difference in size in all experimental groups (i.e., control material, 1 Hz, and 80 Hz) compared with the negative controls.

## 4. Discussion and Conclusions

In this paper, we have introduced the design, fabrication, and validation of a biocompatible piezoelectric-based device for the study of the effects of dynamic mechanical stimulation on cells. The system allows for actuating on the cells in a wide range of frequencies that have been previously identified of relevance in biological processes [18,56,57,58]. Moreover, due to the sinusoidal nature of the induced movement, we can straightforwardly estimate the actual force actuating on the cells, which, in our experiment, remain in the typical ranges analyzed in other experiments [22,59].

Using such devices in cell cultures, we have also made some preliminary studies on the influence of dynamic mechanical stimulations in the HaCaT cell line. We have studied the influence of such stimuli in HaCaT proliferation, migration, and evaluated morphological changes associated with different frequency and magnitude of dynamical stimuli.

Thus, application of 1 Hz frequency stimuli in HaCaT cells seems to improve conditions for wound healing (faster proliferation and migration rates). Cells, on average, retained the same nucleus size as the control ones, increased filopodia projections, and generated a sparser shape. These results suggest that mechanical vibration of low amplitude (nanometer range), long duration (more than 6 days), and low frequency (1 Hz) facilitate the proliferation and motility in vitro of the HaCaT cell line, but compromise cell morphology. Other studies have already demonstrated that mimicking physiological conditions in vitro improve the cellular behavior, entailing cellular proliferation and cell division [58,60,61].

On the other hand, application of an 80 Hz frequency stimulus seems to produce the contrary effect: the proliferation and migration response was decreased, and cell morphology was mostly rounded and smaller compared with controls. As per SEM micrographics, at 48 h, 80 Hz samples seem to be more detached from the ground at their central points, while the control and static device cells retained flat and polygonal. This may indicate that a range of frequency (around 80 Hz) limits the proliferative and migratory response of HaCaT cell line and considerably affects their shape.

Finally, we have used immunofluorescence to better understand the morphological changes observed in SEM experiments. We observed that f-actin was stretched and extended in 1 Hz stimulated samples compared with the static controls at 48 h of continuous stimulation. In 80 Hz stimulated samples, f-actin showed an apparent rounded distribution (along the borders of the cells), but there were insignificant changes compared with controls and static conditions.

As a conclusion, a unique device consisting of a piezoelectric membrane and an electronics stage was described and tested as a stimulus of the mechanotransduction in epithelial cell cultures. The device allows influencing the cell phenotype and behavior without using chemicals based on dynamic simulations of low-scale amplitude. Modifying and controlling the cellular interactions (with simple systems and chemicals-independent) could provide advantages in many research fields such as regenerative-tissue engineering, drug discovery, and biotechnology. For instance, there are no limitations in using our device in different types of cells or cell lines and, therefore, the device can be used for studying the responses of stem cells to dynamic mechanical stimulation. Previous studies have exploited the use of mechanical stimulation in stem cells [21,61]. In their studies, high frequencies (500 Hz and 1 kHz) applied to mesenchymal human stem cells were found to stimulate osteogenesis. As an example, those frequencies can be adjusted in our device.

Finally, our biocompatible device distinguishes for an easy to handle manipulation in in vitro studies, a scalable fabrication, and a dynamic performance that can be varied by the users dependent on the cellular processes under interest. The latter characteristic is impossible in most of the current mechanical stimulation techniques (e.g., scaffolds and substrate patterning, where the same cellular substrate’s properties are fixed during the whole experiment). By testing the device, we have also shown initial findings in two relevant cellular functions (migration and proliferation) of 2D skin tissues, which are essential in the proliferative phase in wound healing. This paves the way for more research with this device and its eventual application on a clinically relevant scenario.

## Figures and Tables

**Table 1:** Summary of the obtained scratch assay parameters.

	Control (C)	Piezoelectric Device w/o Stimulation (CM)	Piezoelectric Device with 1 Hz Frequency Stimulation	Piezoelectric Device with 80 Hz Frequency Stimulation
Slope	−0.03703 ± 0.00102	−0.03614 ± 0.001644	−0.04004 ± 0.002576	−0.03183 ± 0.002552
Total closure time (hours)	27.76	28.25	24.41	33.97

## Appendix A

### A1. Theoretical Analysis of the PVDF Actuator

Polyvinylidene fluoride (PVDF) is a synthetic fluoropolymer with a principal Carbon chain surrounded by Fluor and Hydrogen atoms. There are four known different crystalline phases of PVDF, named α, β, γ, and δ. Owning to the atom’s distribution, the piezoelectricity of the PVDF stems from its β-phase in which all Fluor atoms are found on one side of the Carbon chain and all Hydrogens on the other, resulting in the highest net dipolar moment among its crystalline phases. As for most piezoelectric materials, PDVF needs to be pooled by a large electrical field in a particular direction to provide a piezoelectric effect. The direction of positive polarization is conveniently chosen to coincide with the *z*-axis of a rectangular system, which alternatively is denoted numerically by 1, 2, and 3 representing length or stretch direction, width or transverse direction, and thickness direction, respectively. As such, the poling axis always coincides with axis 3. An external electric field applied to the poling direction will exert a force between the centers of positive and negative charges sites in each domain chain, leading to an elastic strain and hence changes of dimensions in the material. This mechanical deformation when an electric field is applied is called the inverse effect of piezoelectricity. The constitute equation that relates the total strain and both the applied electric field, and any mechanical stress is shown in (A1).

(A1) $$\left[\begin{array}{c} s_{1}\\ \vdots \\ s_{6} \end{array}\;\right]=\left[\;\begin{array}{ccc} d_{11} & \cdots & d_{31}\\ \vdots & \ddots & \vdots \\ d_{16} & \cdots & d_{36} \end{array}\;\right]\cdot \left[\;\begin{array}{c} E_{1}\\ E_{2}\\ E_{3} \end{array}\;\right]+\left[\;\begin{array}{ccc} S_{11} & \cdots & S_{16}\\ \vdots & \ddots & \vdots \\ S_{61} & \cdots & S_{66} \end{array}\;\right]\cdot \left[\;\begin{array}{c} T_{1}\\ \vdots \\ T_{6} \end{array}\;\right]$$

where $s_{i\;}$ is the strain vector, $d_{\mathrm{ij}}$ is the piezoelectric coefficient matrix (tensor), $E_{i}$ is the electrical field, $S_{\mathrm{ij}}$ the compliance matrix (tensor), and $T_{i}$ the stress vector. The terms $T_{1}$ through $T_{3}$ are the normal stresses along axes 1, 2, and 3, whereas $T_{4}$ through $T_{6}$ are shear stresses. Equation (A1) consists of two terms. The first term represents the strain generated by the inverse piezoelectric effect. If there was no mechanical stress present, the second term is zero and the strain is related to the electric field by the first term of Equation (A1). When a homogenous electrical field is applied along axis 3, the deflection in the polarization direction of the piezo is obtained from (A1) by multiplying by its thickness, which is equal to the distance between the electrodes.

The design of the cellular actuator device (Figure 2) can be viewed as a rectangular flat membrane with short edges a, and long edge b, and thickness h—all edges are clamped. The governing differential equation for membrane displacement under uniform stress is [62]:

(A2) $$\frac{\partial^{4}w(x,y)}{\partial x^{4}}+2\frac{\partial^{4}w(x,y)}{\partial x^{2}\partial y^{2}}+\frac{\partial^{4}w(x,y)}{\partial y^{4}}+\frac{{\rho h\omega }^{2}}{D}w(x,y)=0$$

where w(x,y) denotes the normal displacement for a point at a location (x, y), ρ is the mass density, and ω is the angular frequency. The term D represents the rigidity of the plate and is related to E (Young’s modulus), ν (Poisson’s ratio,) and h according to:

(A3) $$D=\frac{{\mathrm{Eh}}^{3}}{{12(1-\nu }^{2})}$$

From the theory of structural mechanics, a solution of (A2) for the fundamental resonant frequency for rectangular membranes with fixed boundaries may be expressed as:

(A4) $$f_{00}=\frac{k_{1}}{2\pi }\sqrt{\frac{\mathrm{Dg}}{{\mathrm{wa}}^{4}}}$$

where $k_{1}$ is a tabulated value for various ratios of ab and the rest of the terms were already defined. Under the assumption of no external force acting on the membrane, the deflection of the piezo is generated by an external electric field that is uniformly distributed along the electrode layer in the polarization direction, and the uniform load w per unit area stands for the inverse piezoelectric effect within the elastic regime of the material; the first natural frequency in (A4) is calculated to be 148 kHz. The vibrational modes of the membrane are integer numbers of the first natural frequency, and, below such a frequency, as it will be the case of our experiments, the displacement on the piezoelectric membrane may be considered within the static and low-frequency range [63]. Parameters of the PVDF used for this calculation are collected in Table A1.

**Table A1:** Parameters of the PVDF films and values for the calculation of the fundamental frequency.

Parameter			Value			
Thickness			28 µm			
Piezoelectric coefficient d_31_			23∙10^−12^ (C/m^2^)/(N/m^2^)			
Piezoelectric coefficient d_33_			−33∙10^−12^ (C/m^2^)/(N/m^2^)			
Young’s modulus			2 to 4 (GPa) or (N/m^2^) [64]			
Poisson’s ratio			0.326			
Mass density			2.10∙10^3^ (kg/m^3^) [65]			
Short edge of the membrane			25 mm			
Long edge of the membrane			35 mm			
a/b	1	0.9	0.8	0.6	0.4	0.2 [63]
k_1_	36	32.7	29.9	25.9	23.6	22.6
